# Chemical Characterization, Antioxidant and Antimicrobial Properties of Different Types of Tissue of *Cedrus brevifolia* Henry Extracts

**DOI:** 10.3390/molecules27092717

**Published:** 2022-04-22

**Authors:** Despina Charalambous, Nicolas-George Homer Eliades, Michalis Christoforou, Eleni Kakouri, Charalabos Kanakis, Petros A. Tarantilis, Maria Pantelidou

**Affiliations:** 1 Department of Pharmacy, School of Health Sciences, Frederick University, 7, Y. Frederickou Str., Pallouriotissa, Nicosia 1036, Cyprus; hsc.cd@frederick.ac.cy (D.C.); mic.christoforou@gmail.com (M.C.); 2 Nature Conservation Unit, Frederick University, Nicosia 1036, Cyprus; res.en@frederick.ac.cy; 3 Laboratory of Chemistry, Department of Food Science & Human Nutrition, School of Food and Nutritional Sciences, Agricultural University of Athens, Iera Odos 75, 118 55 Athens, Greece; elenikakouri@aua.gr (E.K.); chkanakis@aua.gr (C.K.); ptara@aua.gr (P.A.T.)

**Keywords:** *Cedrus brevifolia*, Cyprus, antioxidant, antibacterial, coniferous, LC/Q-TOF/HRMS, phenolic compounds

## Abstract

This study aimed to determine the chemical composition of different types of tissue of *Cedrus brevifolia* Henry (Pinaceae) methanolic extracts, namely needles, twigs, branches, and bark. *Cedrus brevifolia* is a narrow endemic coniferous tree species of Cyprus, growing in a sole population in the mountainous area of Paphos Forest. Chemical analysis of the extracts was performed using liquid chromatography combined with time-of-flight high-resolution mass spectrometry (LC/Q-TOF/HRMS). The majority of the 36 compounds tentatively identified belonged to the flavonoids family. The extract of needles was the richest extract in terms of secondary metabolites. The extracts were studied for their antioxidant activity using the DPPH free radical scavenging assay. Additionally, the antibacterial activity was evaluated by determining both the minimum inhibitory concentration and the minimum bactericidal concentration against *Staphylococcus aureus* and *Escherichia coli*. All extracts demonstrated antioxidant property, while bark gave the highest antioxidant capacity (IC_50_ value of 0.011 mg/mL) compared to the other tissues. Antibacterial activity was observed against both types of bacteria, with the extract of branches presenting the strongest activity against *S. aureus* (MIC, 0.097 mg/mL and MBC, 0.195 mg/mL). This is the first time that extracts of needles, twigs, branches, and bark of *C. brevifolia* are compared regarding their chemical composition as well as their antimicrobial and antioxidant properties.

## 1. Introduction

*Cedrus brevifolia* (Hook. F.) A. Henry, also known as the Cyprus cedar, is a narrow endemic coniferous tree on the island of Cyprus and is one of the four species of the *Cedrus* genus [1]. Based on morphological and ecophysiological traits, *C. brevifolia* is differentiated from other species of the same genus, characterized by short needles [1,2], resistance to aphids [3], and tolerance to drought [4]. Despite the lack of paleontological data for *C. brevifolia*, several studies have suggested that the species has had a long history in Cyprus. Theophrastus (371–287 B.C.) was the first to mention the presence of cedar in Cyprus [5]. It is estimated that the time of divergence between *C. libani* and *C. brevifolia* (molecular clock) was between 7.83 (±2.79) to 6.56 (±1.20) million years ago [6]. Today the tree grows in a sole population in the Paphos forest in an area of 269 ha, in an altitudinal zone of 900–1400 m [7]. The species has a specific conservation status, as it is classified as vulnerable according to the International Union for Conservation of Nature Red List of Threatened Species [8], while its habitat is included in Annex I of the European Directive 92/43/EEC (The Habitats Directive), characterized as a priority habitat type in Europe [9].

In ancient times, the timber of *C. brevifolia* was periodically subjected to intense exploitation for ship construction, furniture purposes, and especially for woodcut icon screens and other woodwork in churches [10]. According to oral tradition, during the past century, the wood of Cyprus cedar was not exclusively regarded as a forest product but rather as wood of exquisite quality, aromatic and resistant to insects and fungal infections [10]. As previously reviewed [11], several studies have demonstrated that extracts from cedar species have been used in cosmetics, foods, and a variety of household products since ancient times. Furthermore, the essential oil from cedar species was found to demonstrate antibacterial, antioxidant, and antifungal properties [11]. Studies have shown that the extract from *Cedrus* species is pharmacologically active as it possesses anticancer and anti-proliferative properties against human K562 chronic myelogenous leukemia cells [12].

Although extracts from three of the four *Cedrus* species (*C. libani*, *C. atlanctica*, and *C. deotara*) have been studied extensively, the narrow endemic *C. brevifolia* has not been widely studied. Research on crude extracts from *C. brevifolia* bark has shown that the phenolic content demonstrates in vitro antioxidant and anti-inflammatory activity [13]. Additionally, according to the literature, methanol and methanol:water extracts from C. *brevifolia* needles presented high reducing activity [14]. It was later suggested that the characteristic and rare odor in *C. brevifolia* is attributed to 4-acetyl-1-methylcyclohexene, a compound found in its heartwood in a volume of 1.1% [15]. More recently, a study demonstrated that the essential oil from the needles has antioxidant and antimicrobial properties against bacteria and fungi [16].

The investigation of the phytochemicals of plants for determining their pharmacological properties and thus their usefulness in tackling disease continues to be of great interest to the scientific community. To this date, many plant species are being investigated for their chemical composition using different extraction or analysis methods to study their antioxidant and antimicrobial properties, e.g., [17,18,19,20]. More information on the phytochemical profile and properties of *C. brevifolia* remains to be determined using different extraction methods. Additionally, data regarding the antioxidant and antimicrobial activity of different types of tissue of *C. brevifolia* are not extensively available. Taking into consideration the importance of endemic species along with their potential use for their pharmaceutical properties, this study aims to present new data regarding the qualitative chemical profile of different types of tissue of *C. brevifolia* methanolic extracts and also investigate their antioxidant and antimicrobial properties.

## 2. Results

### 2.1. Chemical Analysis of Methanol Extract of C. brevifolia

The extracts of *C. brevifolia* were analyzed under the negative ionization mode using LC/Q-TOF/HRMS analysis, as described in the Materials and Methods section. Compounds tentatively identified are presented in Table 1. Information regarding the *m/z* values of the deprotonated ions, retention time (t_R_), and ms/ms product ions are also shown. The abundance of compounds that mostly belong to the flavonoid family, either glycosylated or not, is notable. Needle extract was the richest as far as the number of compounds identified, whereas the extract of branches showed a simpler chemical profile. Overall, 36 compounds were detected, while at the same time qualitative differences between the tested extracts are not negligible.

### 2.2. Determination of Total Phenolic Content (TPC)

The total phenolic content of methanol extracts of needles, twigs, branches, and bark was determined using the Folin–Ciocalteu method [24]. A standard curve of gallic acid was constructed and the results are expressed as mg gallic acid equivalent of *C. brevifolia* methanol extracts per gram of crude extract (mg GAE/g). According to the data, all extracts (needles, twigs, branches, and bark) contained phenols at concentrations ranging from 16.656 to 38.405 mg GAE/g crude extract (Table 2). The bark methanol extract, demonstrated the highest total phenol content with a TPC value of 38.405 mg GAE, followed by twigs (29.726 mg GAE), branches (17.980 mg GAE), and needles methanol extract (16.656 mg GAE).

### 2.3. Antioxidant Activity of Methanol Extracts of C. brevifolia

Crude methanol extracts from needles, twigs, branches, and bark were examined for their antioxidant activity and results were compared with Trolox, a known antioxidant. 1,1-diphenyl-2-picryl-hydrazyl (DPPH) radical scavenging activity (RSA) was carried out to evaluate the free radical scavenging effect of the extracts. Results were expressed as the half maximum inhibitory concentration (IC_50_) of *C. brevifolia* methanol extracts, which is defined as the concentration of each sample (mg/mL) required to scavenge DPPH radical by 50% [25]. The Trolox equivalent antioxidant capacity (TEAC) was calculated to determine the antioxidant capacity of the samples in comparison to Trolox, as previously described [25]. According to our data, all extracts (needles, twigs, branches, and bark) exhibited significant antioxidant activity (Table 3). However, the methanol extract of bark demonstrated the most potent DPPH RSA activity with an IC_50_ value of 0.011 mg/mL, followed by the methanol extract of twigs (0.031 mg/mL), branches (0.062 mg/mL), and needles (0.078 mg/mL). The TEAC value of all extracts was calculated and the results are presented in Table 3. Similarly, the methanol extract of bark demonstrated the highest TEAC value (98%), followed by the extracts of twigs, branches, then needles.

### 2.4. Antimicrobial Activity of Extracts

#### 2.4.1. Minimum Inhibitory Concentrations and Minimum Bactericidal Concentration against *S. Aureus* and *E. Coli* Bacteria

The minimum inhibitory concentration (MIC) and minimum bactericidal concentration (MBC) of methanol extracts of needles, twigs, branches, and bark were evaluated against Gram-negative bacteria, *E. coli*, and Gram-positive bacteria, *S. aureus.* A low MIC value indicates that less extract is needed to inhibit the growth of bacteria. As shown in Table 4, all extracts demonstrated strong bacterial inhibition activity, with MIC values ranging from 0.097–0.781 mg/mL against *S. aureus*, whereas the inhibition activity against *E. coli* was weaker (3.125–6.25 mg/mL). Interestingly, the branch methanol extract exhibited the highest bacterial inhibition activity (MIC, 0.097 mg/mL), whereas the extract from needles demonstrated the lowest inhibition activity (MIC of 0.781 mg/mL) against *S. aureus*. All extracts demonstrated a weaker activity against *E. coli* compared to *S. aureus*, with branch and needle extracts showing the lowest inhibition activity (MIC, 6.25 mg/mL for both).

The antimicrobial efficacy was also determined in terms of MBC, which defines the lowest concentration of the extract that has bactericidal activity. Therefore, the lower the MBC value, the lower the quantity of extract needed to kill bacteria. MBC values are shown in Table 4. All extracts demonstrated bactericidal activity, with MBC values ranging from 0.195–0.781 mg/mL for *S. aureus* and 6.25–12.5 mg/mL for *E. coli*, suggesting that *S. aureus* is more susceptible to the extracts. Again, the branch methanol extract exhibited the highest bactericidal activity (MBC, 0.195 mg/mL) compared to other tissues. The ratio MBC/MIC was evaluated in order to determine bactericidal (kills bacteria) or bacteriostatic (inhibits growth) activity. A ratio MBC/MIC of ≤4 is measured as bactericidal, while an MBC/MIC ratio of >4 is considered bacteriostatic [28]. As shown in Table 4, the MBC/MIC ratio values of all extracts demonstrated bactericidal activity (MBC/MIC ≤ 4).

#### 2.4.2. Time–Kill Assay

**Figure 1:**
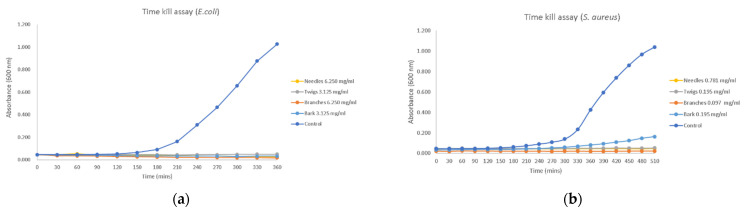
Time–kill assay for *C. brevifolia*: (**a**) Growth curves of *E. coli* with methanol extracts of needles, twigs, branches, and bark; (**b**) Growth curves of *S. aureus* with methanol extracts of needles, twigs, branches, and bark.

A separate time–kill assay was conducted for each bacterial strain (*E. coli* and *S. aureus*) over a period of 6–8 h following inoculation. The results are shown in Figure 1. According to these findings, at extract concentrations corresponding to MIC, no measurable bacterial growth was observed for any of the bacterial strains in the first 30 min after inoculation, and bacterial growth was completely blocked after 6 h. All of these inhibition patterns remained unchanged until the end of the time course (6 h) with an average of 97% dead bacterial cells.

## 3. Discussion

To our knowledge, this is the first report comparing the chemical profile as well as the antimicrobial and antioxidant properties of methanol extracts of different types of tissue of *C. brevifolia* grown in Cyprus. More specifically, methanol extracts of needles, twigs, branches, and bark were studied, and the chemical compounds of each type of tissue were identified using LC/Q-TOF/HRMS analysis. The identification of active compounds from natural sources can be achieved through a variety of analytical techniques. Even though LC/Q-TOF/HRMS analysis does not give information on the chemical structure or the type of sugar attached to the aglycon part of a compound, no strong limitation concerning the identification of the eluted compounds exists. LC/Q-TOF/HRMS analysis permits accurate mass measurement that increases the accuracy of the predicted chemical formula. In addition, observed product ions that are generated from the MS/MS analysis, in combination with the literature data, provide results of high reliability [29].

Several studies deal with the chemical analysis of *Cedrus* species and their biological activity. Nevertheless, many of them focus on the essential oil obtained from different parts of the tree [11,15]. The present work aimed to identify the polar metabolites from different tissues of the tree, as reported scientific data according to current research are insufficient. A study by Douros et al. [14] examined the chemical profile of the polar fraction of *C. brevifolia* needles. The authors analyzed methanol and methanol:water extracts and discussed the presence of phenolic compounds. Compared to our results, there are some common compounds identified in both studies; however, major differences are observed. This can be explained taking into account the different solvents used for the extraction and possibly the different location of the tree; although both were found in Paphos forest, this state forest has an extent of approximately 269 ha. In another study performed by Cretu et al. [13], the bark of the tree was analyzed for its chemical profile. The authors reported taxifolin, its glucosides, catechin and epicatechin, as well as procyanidin oligomers, as the main constituents. Among the *Cedrus* species, *C. deodara* has been studied extensively. *C. deodara* is native to the Himalayas and its reported chemical profile, as far as the flavonoid and lignan content, resembles that of the species presented here [21,30]. Our results are in accordance with the studies mentioned above, but it also reports compounds that have not been previously identified in *C. brevifolia*. Precisely, compounds **1**, **5**, **6**, **7**, **11**, **13**, **15**, **17**, **18**, **21**, **23**, **26**, **29**, **31**, **32**, **33**, and **34** are reported here for the first time as chemical constituents of *C. brevifolia*. In addition, qualitative differences between the chemical profile of the extracts tested are notable.

As it has been reported, needles seem to have considerable antioxidant activity [14,16]. Although the results of our study confirm this, they also demonstrate that the other extracts have a notable antioxidant activity, with the extract of bark being the most potent. Taxifolin (compound **8**), a known antioxidant, was identified at the extracts of bark and branches. The same applies for deodardione (compound **36**), a sesquiterpene. On the other hand, compounds **2**, **6**, **20**, **22**, and **33** were only detected in the extracts of barks and twigs. Given these data, the difference between the antioxidant activity of the extracts could be attributed not only to differences in their chemical composition, but also to a possible synergistic effect between the compounds detected, as previously suggested by other studies [31,32]. However, whether composition differences and/or similarities influence the antioxidant activity of the extracts, remains to be clarified. Nonetheless, according to our experiments, all extracts demonstrated a high phenolic content compatible with their antioxidant activity, while the methanol extract of bark demonstrated the highest phenolic content and the most potent DPPH RSA (IC_50_, 0.011 mg/mL). This is confirmed with TEAC values which were calculated by comparing antioxidant activity of each extract to Trolox. These data suggest that the bark is the primary source of antioxidant constituents in *C. brevifolia*, a finding which is in agreement with previously reported data on the antioxidant activity of crude extracts and the phenolic content of the bark of *C. brevifolia* [13,33].

Further to the antioxidant activity, all extracts were assessed for their antibacterial activity. This was assessed by estimating MIC and MBC. The ratio MBC/MIC value was determined in order to identify bactericidal versus bacteriostatic properties. According to MBC/MIC values, all extracts demonstrated bactericidal activity against *S. aureus* and *E. coli*. The methanol extract of branches exhibited the highest inhibition of growth and bactericidal activity against *S. aureus*, a finding also confirmed by time–kill experiments. Taxifolin (compound **8**) identified in the extracts of branches and bark has been previously reported to demonstrate an antibacterial effect on *S. aureus* [34]. Taxifolin belongs to flavanones. As for all flavonoids, flavanones’ antimicrobial activity is related to their structure. In particular, regarding taxifolin, the presence of hydroxyl groups at positions 5 and 7 (A ring), 5′ (B ring), and the saturated 2–3 bond, are crucial for enhancing the antimicrobial activity. It is also worth mentioning that isosakuranetin, a methoxy derivate of naringenin that was only detected in the extract of branches (compound **34**), presents in general a moderate antimicrobial activity. This is attributed to the presence of a methoxy group at position 4′ (B ring) [35,36]. Furthermore, himaphenolone (compound **35**) and deodardione (compound **36**), two sesquiterpene ketones, were also tentatively identified in the extracts, with the latter being detected only at the extracts of branches and bark. Terpenes, and their derivatives sesquiterpenes, are well known for their antimicrobial activity against Gram+ and Gram-bacteria [37,38,39].

In conclusion, plant extracts are chemically complex mixtures which seem to possess a variety of biological activities. Secondary metabolites produced by plants, specifically polyphenols such as flavonoids, are known for their antioxidant [40] and antibacterial activity [41]. There is a common consensus that naturally derived compounds found in extracts usually present a synergistic biological effect [42,43]. In this regard, plant extracts may be useful as coadjuvant agents against a human multifactorial disease, thus the search of new biologically active natural sources is in constant demand. Our study contributes to this continuous attempt as it presents new data regarding the chemical analysis of *C. brevifolia* which consequently highlight its antioxidant and antimicrobial properties. Considering that this species has not been studied as extensively as the other three cedar species (*C. libani*, *C. atlantica*, and *C. deotora*), the results of this study make a significant contribution to the knowledge regarding the Cyprus cedar. Specifically, the composition of different types of tissue of the tree (needles, twigs, branches, and bark) was tentatively identified, and comparisons were made regarding the antioxidant and antibacterial properties of each type. Further assessment of the single chemical compounds and the study of potential synergistic effects will possibly help better understand their properties.

## 4. Materials and Methods

### 4.1. Plant Material

For the purposes of the present study, sampling of 12 twigs (20–30 cm long each, two twigs from each of six different trees of *C. brevifolia*) and a branch was carried out during the implementation of silvicultural interventions in *C. brevifolia* stands through the LIFE-KEDROS project (LIFE15 NAT/CY/00850). All samples were collected from the main area of the species occupancy in Tripylos mountain. Sampling was carried out within a surface of 1 ha with the coordinates of the central point of this surface being as follows: x: 470,618.63; y: 3,872,738,56; z: 1365 (in UTM system 36S). To further investigate the antimicrobial and antioxidant properties of methanol extracts, the sampled tissues were separated into four types of tissue: (i) needles (200–300 g), collected from each sampled twig, (ii) twigs (200–300 g), after their separation from the needles, (iii) bark (200–300 g), from the sampled branches, with the cambium also included in this sample, and (iv) branches (200–300 g), including both heartwood and sapwood. All samples were kept at 4 °C until further analysis.

### 4.2. Preparation of Extracts

Cyprus cedar extracts of needles, twigs, branches, and bark were dried and then crushed into powder with a mixer. Powder (10 g each time) was added to 150 mL solvent (100% methanol, Merck) at room temperature for 24 h. Thereafter, the extracts were centrifuged at 25 °C, 3000 rpm for 10 min, filtered, and condensed using a rotary evaporator (Stuart RE300, Keison, UK) at 45 °C under vacuum. All filtrates were stored at 4 °C for further analysis. Yield of the different extracts ranged between 6.5–13.9 g extract/100 g dried extract.

### 4.3. LC-Q-TOF/HRMS Analysis

Crude extracts were dissolved in methanol (LC-MS grade, Sigma Aldrich, Taufkirchen, Germany). Standard solutions of catechin, quercetin, naringenin, kaempferol, and isorhamnetin were similarly prepared. All standard solutions were purchased from Extrasynthese (Genai, France). All samples were prepared the day of the analysis. Chemical analysis was performed on a HPLC system (Agilent Series 1260, Agilent Technologies, Santa Clara, CA, USA), equipped with a degasser, autosampler, quaternary pump, diode array, detector, and column oven, coupled to a 6530 Q-TOF mass spectrometer (Agilent Technologies, Santa Clara, CA, USA). The LC analysis was performed on a EC nucleoshell Bluebird RP18, 4.6 mm × 100 mm, 2.7 μm column at 30 °C, and the solvent system consisted of ultrapure water (Genie A ultrapure water system, Rephile Bioscience, Miami, FL, USA) (solvent A) and acetonitrile (Sigma Aldrich, Taufkirchen, Germany) (solvent B), both acidified with 0.1% acetic acid LC-MS grade (Fisher Scientific, Hampton, NH, USA). The elution program was the following: 10–90% (B) for 0–8 min, 30–70% (B) from 8–12 min, 40–60% (B) from 12–16 min, 50–50% (B) from 16–18 min, and then maintained at 10% (B) up to 33 min. Chromatograms were recorded at 280, 320, 330, 360, and 520 nm. Flow rate was adjusted to 1 mL/min and the injection volume was 10 μL. The Q-TOF mass spectrometer was operated at the negative ionization mode according to the following parameters: capillary voltage 4000 V, gas temperature 300 °C, skimmer 65 V, octapole RF 750 V, drying gas 10 L/min, nebulizer pressure 450 psig, and fragmentor voltage 150 V. The *m*/*z* measure ranged from 100–1600. The CID-MS/MS spectra were recorded on the auto ms/ms mode and the *m*/*z* range was set to 50–800. Collision energy was set at 50 V. Data were analyzed using the Agilent MassHunter Workstation Software LC-MS data Acquisition.

### 4.4. Total Phenolic Content

The total phenolic content of *C. brevifolia* methanol extracts was determined by using the Folin–Ciocalteu method as described by Shirazi et al. (2014). A standard gallic acid (Sigma Aldrich, Taufkirchen, Germany) curve was constructed by preparing dilutions of 0.05–0.4 mg/mL in methanol. A total of 100 μL of each of these dilutions were mixed with 500 μL water and then 100 μL of Folin–Ciocalteu reagent (Sigma Aldrich, Taufkirchen, Germany) and allowed to stand for 6 min. Then, 1 mL of 7% sodium carbonate (Sigma Aldrich, Taufkirchen, Germany) and 500 μL distilled water was added to the reaction mixture. The absorbance was recorded after 90 min at 760 nm spectrophotometrically (UV-1280, Shimadzu Europa GmbH, Duisburg, Germany). The same procedure was repeated with *C. brevifolia* methanol extracts. The total phenolic content of *C. brevifolia* methanol extracts was calculated as gallic acid equivalents (mg GAE/g crude extract) using the linear regression equation of the gallic acid standard curve. All experiments were performed in triplicate and the results were expressed as the mean value ± standard deviation (SD).

### 4.5. Antioxidant Activity

Antioxidant activity of *C. brevifolia* extracts was determined using the DPPH free radical-scavenging assay as previously described [44]. The starting concentration of the extracts was 100 mg/mL and 2-fold serial dilutions were performed. DPPH solution (0.5 mM in 95% ethanol, Sigma Aldrich, Taufkirchen, Germany) was used and the absorbance was recorded at 515 nm using a microplate reader (Sunrise, Tecan Trading Ltd., Männedorf, Switzerland). Trolox (Sigma Aldrich, Taufkirchen, Germany) was used as a reference standard and a standard calibration curve with sequential concentrations was prepared following the same procedure. Results were expressed as the mean value of three independent experiments (±SD). The degree of decolorization indicated the free radical scavenging efficiency of the extracts. The antioxidant activity of *C. brevifolia* extracts was calculated as % scavenging activity of the DPPH solution. The capability to scavenge the DPPH radical was calculated using the following equation: DPPH Scavenged (%) = ((AB − AA)/AB) × 100 (AB is the absorbance of control sample and AA is the absorbance of the sample at 30 min). The IC_50_ of the standard and the *C. brevifolia* extracts were defined as the concentration of the extracts (mg/mL) required to scavenge the DPPH radical by 50%. The TEAC of the extracts was calculated to determine the antioxidant capacity as compared to the standard, Trolox. TEAC value was calculated as follows: TEAC = IC_50_ of Trolox (mg/mL)/IC_50_ of sample (mg/mL). The higher TEAC value means the higher DPPH RSA.

### 4.6. Antibacterial Activity

#### 4.6.1. Determination of MIC

The antibacterial activity of the *C. brevifolia* methanol extracts was studied by determining the MIC via the broth micro-dilution method. Aliquots of each *C. brevifolia* methanol extract were transferred in a 96-well plate. Specifically, 200 μL of each extract (50 mg/mL) were added as a starting solution and 2-fold serial dilutions with Tryptic Soy broth (TSB) were prepared (Liofilchem, Roseto degli Abruzzi, Italy). Isolated cultures of *E. coli* (NCTC 9001) and *S. aureus* (NCTC 6571) (Sigma Aldrich, Taufkirchen, Germany) were prepared in TSB at a concentration of about 1 × 10^6^ cfu/mL. One-hundred microliters (100 µL) of each bacterial inoculum were added in each well, containing either extract or controls. Blank samples of each extract (containing no bacteria) were subjected to 2-fold serial dilution with TSB (blank control). Control samples including bacteria (100 uL) but no extract were used as growth controls. Additionally, a sterility control was used with TSB, no bacteria, and no extract. Wells with bacteria and Ampicillin (0.516 mg/mL, Sigma Aldirch, Taufkirchen, Germany) or Gentamycin (0.064 mg/mL, Molekula, Durham, UK) were used as positive controls. The MIC of each sample was detected after 18h of incubation at 37 °C, following the addition (30 µL) of 0.2 mg/mL p-iodonitrotetrazolium chloride (INT) (Sigma Aldrich, Taufkirchen, Germany) and incubation at 37 °C for 30 min. The absorbance of each plate was measured at 492 nm with a microplate reader (Sunrise, Tecan Trading Ltd., Männedorf, Switzerland). As viable bacteria reduce the yellow dye to pink, MIC of each extract was defined as the sample concentration that prevented the color change of the medium and exhibited complete inhibition of bacterial growth as compared with that of the blank control.

#### 4.6.2. Determining MBC

The MBC of the methanol extracts was determined by sub-culturing 2 μL aliquots of the preparations from the MIC assay in 100 μL TSB and incubating for 24 h at 37 °C. The MBC was defined as the lowest concentration of each sample which did not exhibit a color change after addition of INT as described above.

#### 4.6.3. Time–Kill Assay

*Cedrus brevifolia* methanol extracts were tested for their time–kill behavior against *E. coli* and *S. aureus*. In a 96-well plate, 200 μL of *C. brevifolia* samples (50 mg/mL) were added along with 100 μL Tryptic Soy Broth (TSB). Each extract was subjected to 2-fold serial dilution with TSB. An aliquot of 100 μL of overnight grown bacterial culture (*E. coli* or *S. aureus*) was added to each column at a final concentration of 1 × 10^5^ cfu/well. A blank column was also prepared for each extract with a 2-fold serial dilution with TSB, thus for measuring the background absorbance of each sample solution. The plates were then incubated at 37 °C and optical density was recorded at 30 min intervals at a wavelength of 600 nm until the cells reached the stationary phase using a microplate reader (Sunrise, Tecan Trading Ltd., Männedorf, Switzerland), linked to a computer equipped with Magellan 7.5 software. A graph of the absorbance (nm) against time (minutes) was plotted for each sample. A growth control containing only bacteria (no extract) and a sterility control containing only TSB and methanol were also used. Ampicillin (0.516 mg/mL, Sigma Aldrich, Taufkirchen, Germany) and Gentamicin (0.064 mg/mL, Molekula, Durham, UK) were used as positive controls.

## Figures and Tables

**Table 1:** Chemical composition of methanol extracts of different types of tissue of *C. brevifolia* tentatively identified by LC/Q-TOF/HRMS analysis.

Compound Number	t_R_	Molecular Formula	Observed ion (*m/z*) [M-H]^−^	ms/ms Productions	Compound Name	Twigs	Needles	Branches	Bark	Reference
**1**	1.72	C_14_H_18_O_9_	329.0874	167.0346 152.0110 123.0085	vanilloyl hexoside	+	+	-	+	NA
**2**	1.89	C_13_H_16_O_8_	299.0769	137.0239 121.0282 119.0363	hydroxybenzoic acid hexoside	+	-	-	+	[14]
**3**	2.83	C_16_H_20_O_9_	355.1023	297.0569 193.0506 119.0475	ferrulic acid hexoside	+	+	-	+	[21]
**4**	3.26	C_15_H_14_O_6_	289.0713	SS	catechin	+	+	-	+	[14]
**5**	3.59	C_21_H_22_O_13_	481.0972	463.085 289.0711 163.0348	epigallocatechin glucuronate	+	-	-	-	NA
**6**	5.45	C_21_H_22_O_12_	465.1026	447.0897 303.0504 151.0035	quercitrin hydrate	+	-	-	+	NA
**7**	5.91	C_22_H_21_O_13_	495.1126	315.0507 163.0349 121.0284	methyl epigallocathechin glucuronate	-	+	-	-	NA
**8**	6.195	C_15_H_12_O_7_	303.0501	285.0381 193.0450 151.0360	taxifolin	-	-	+	+	[14,21]
**9**	6.27	C_15_H_12_O_8_	319.0453	301.0353 245.0408 151.0026	dihydromyricetin	+	+	-	+	[21,22]
**10**	6.92	C_27_H_30_O_15_	593.1492	285.0391 229.1479 151.0001	kaempferol rutinoside	-	+	-	-	[14]
**11**	7.02	C_28_H_32_O_16_	623.1587	315.0511 287.0555 151.0030	isorhamnetin rutinoside	-	+	-	-	NA
**12**	7.12	C_16_H_14_O_8_	333.0606	315.0471 209.0407 166.0245	cedrin	+	+	-	-	[21,22,23]
**13**	7.15	C_16_H_12_O_8_	331.0451	313.0334 271.0621 151.0053	methylmyricetin	+	+	-	-	NA
**14**	7.26	C_21_H_20_O_11_	447.0921	285.0376 241.0491 151.0034	astragalin	-	+	-	-	[14]
**15**	7.36	C_22_H_24_O_12_	479.1183	461.1055 271.0589 137.024	methyl epicathechin glucuronate	+	+	-	+	NA
**16**	7.46	C_22_H_22_O_12_	477.1018	315.0368 299.0541 151.0392	isorhamnetin hexoside	+	+	-	-	[14]
**17**	7.60	C_26_H_32_O_11_	519.1854	357.1317 219.0702 206.0531	matairesinoside	+	+	-	-	NA
**18**	7.78	C_20_H_18_O_10_	417.0817	285.0336 213.0571 151.0029	kaempherol pentoside	-	+	-	-	NA
**19**	7.86	C_23_H_24_O_13_	507.1129	344.0505 271.0181 151.0064	syringetin glucoside	-	+	-	-	[14]
**20**	8.01	C_16_H_14_O_7_	317.0656	300.0569 165.0195 151.0398	deodarin or cedeodarin ^1^	+	-	-	+	[21,22]
**21**	8.66	C_23_H_22_O_12_	489.103	300.0346 151.0069 107.0485	quercetin acetyl pentoside	-	+	-	-	NA
**22**	8.75	C_16_H_14_O_7_	317.0659	300.0557 165.0189 151.0408	deodarin or cedeodarin ^1^	+	-	-	+	[21,22]
**23**	8.88	C_24_H_24_O_13_	519.1128	315.1827 285.0388 151.0007	isorhamnetin acetyl glucoside	-	+	-	-	NA
**24**	9.13	C_20_H_22_O_7_	373.1281	312.1009 237.0729 93.0366	wikstromol	+	+	+	+	[21,23]
**25**	9.19	C_25_H_26_O_14_	549.1233	345.0614 271.0263 151.0037	cedrusone A	-	+	-	-	[23]
**26**	9.84	C_22_H_22_O_12_	477.1035	315.6988 165.0195 121.0285	rhamnetin hexoside	+	-	-	-	NA
**27** ^1^	9.9	C_15_H_10_O_7_	301.0349	SS	quercetin	-	+	-	-	[23]
**28**	10.19	C_30_H_22_O_13_	593.1279	447.0896 307.093 285.1532	tiliroside	+	+	-	-	[14]
**29**	10.38	C_31_H_28_O_14_	623.1387	447.0969 285.0400 151.0044	kaempferol 3-(6″-ferulylglucoside)	-	+	-	-	NA
**30**	11.10	C_20_H_22_O_6_	357.1343	342.1077 123.0436 122.0371	matairesinol	+	+	+	+	[21,23]
**31**	11.22	C_15_H_12_O_5_	271.0606	SS	naringenin	+	-	+	+	NA
**32**	11.84	C_15_H_10_O_6_	285.0412	SS	kaempferol	+	-	-	-	NA
**33**	12.27	C_16_H_12_O_7_	315.0502	SS	isorhamnetin	+	-	-	+	NA
**34**	13.48	C_16_H_14_O_5_	285.0764	271.1185 151.0380 119.0501	isosakuranetin	-	-	+	-	NA
**35**	15.10	C_15_H_22_O_2_	233.1543	217.1204 165.1230 107.0479	himaphenolone	-	+	+	-	[23]
**36**	15.33	C_15_H_22_O_3_	249.1495	149.091 121.1011 68.9963	deodardione	-	-	+	+	[23]

+ presence of the compound; - absence of the compound; NA no available literature data; SS identification according to standard solution; and ^1^ stereoisomers.

**Table 2:** Total Phenolic Content (TPC) of methanol extracts of different types of tissue of *C. brevifolia* using the Folin–Ciocalteu method ^1^.

Tissues of *C. brevifolia*	TPC (mg GAE ^1^/g Crude Extract) ±SD
Needles	16.656 ^c^ ± 1.058
Twigs	29.726 ^b^ ± 2.725
Branches	17.980 ^c^ ± 1.310
Bark	38.405 ^a^ ± 4.687

^1^ mg GAE/g crude extract: mg gallic acid equivalents per g of crude extract; SD: Standard deviation. ^a–c^ Values having different letters differ significantly (*p* < 0.05).

**Table 3:** Antioxidant activity (IC_50_ and TEAC) of methanol extracts from different types of tissue of *C. brevifolia* using the DPPH radical scavenging activity ^1^.

Tissues of *C. brevifolia*	IC_50_ Concentration (mg/mL) ±SD	TEAC % ±SD
Needles	0.078 ^c^ ± 0.007	10.287 ± 0.454
Twigs	0.031 ^b^ ± 0.006	27.630 ± 3.118
Branches	0.062 ^c^ ± 0.008	13.120 ± 1.076
Bark	0.011 ^a^ ± 0.001	97.667 ± 4.041
Trolox (control)	0.009 ± 0.001	-

^1^ Trolox was used as a positive control. Results were expressed as the mean values of three independent experiments. IC_50_ was calculated as previously described [25]. The lower the IC_50_, the higher the antioxidant activity. ^a–c^ Values having different letters differ significantly (*p* < 0.05). IC_50_: half maximum inhibitory concentration; SD: Standard deviation; and TEAC: Trolox equivalent antioxidant capacity.

**Table 4:** Minimum inhibitory concentration (MIC), minimum bactericidal concentration (MBC), and ratio MBC/MIC of *C. brevifolia* methanol extracts against *E. coli* (Gram-negative) and *S. aureus* (Gram-positive) bacteria.

Tissues of *C. brevifolia*	*E. coli*			*S. aureus*		
	MIC ^2^ (mg/mL)	MBC ^3^ (mg/mL)	MBC/ MIC ^4^	MIC ^2^ (mg/mL)	MBC ^3^ (mg/mL)	MBC/ MIC ^4^
Needles	6.250	12.5	2	0.781	0.781	1
Twigs	3.125	6.25	2	0.195	0.390	2
Branches	6.250	12.5	2	0.097	0.195	2
Bark	3.125	12.5	4	0.195	0.390	2
Amp (control) ^1^	0.004	0.004	1	-	-	-
Gen (control) ^1^	-	-	-	0.004	0.008	2

^1^ Ampicillin and gentamycin were used as control antimicrobial agents against *E. coli* and *S. aureus*, respectively; ^2^ The lower the MIC value, the less extract is needed for inhibiting the growth of the bacteria. Compounds with MIC values of <0.6 mg/mL are considered strong inhibitors, 0.6–1.6 mg/mL moderate, 1.6–8.0 mg/mL weak, and >8.0 mg/mL are considered low bacterial inhibitors [26,27]; ^3^ MBC is the lowest concentration of the extract that is bactericidal. The lower the MBC value, the less extract is needed to kill the bacteria; ^4^ Ratio MBC/MIC of ≤4 demonstrates a bactericidal effect, ratio MBC/MIC > 4 demonstrates a bacteriostatic effect [28]. Amp: Ampicillin; Gen: Gentamycin; MIC: Minimum Inhibitory Concentration; and MBC: Minimum Bactericidal Concentration.

## Data Availability

Data reported in this study are contained within the article. The underlying raw data are available on request from the corresponding author.

## Sample Availability

Samples of *C. brevifolia* methanolic extracts are available from the authors.
